# Machining Surface Improvement through Electric- and Flow-Field Adjustments in Flying Electrochemical Milling of AA 2219

**DOI:** 10.3390/ma17040829

**Published:** 2024-02-08

**Authors:** Shukai Fan, Xiaoyun Hu, Junzhi Shen, Xin Ma, Hansong Li

**Affiliations:** College of Mechanical & Electrical Engineering, Nanjing University of Aeronautics and Astronautics, Nanjing 210016, China; arienfan@nuaa.edu.cn (S.F.);

**Keywords:** aerospace aluminum 2219, electrochemical milling, rectangular cathode, cathode design

## Abstract

Electrochemical milling is an ideal technique for machining large-scale 3D structures that consist of aerospace aluminum alloys. The distribution of the electric and flow fields are vital to the quality of the machined surface, and the structures of the inner flow channel and bottom outlet have different effects on the electric and flow fields on the machining surface. In this study, two specialized structures of a tool cathode were optimized by simulating the electric and flow fields, and a reasonable design basis for the tool cathode was obtained. Based on this, an ECM experiment was performed with the same machining parameters using different tools, and a 20 mm × 20 mm plane was machined. The experimental results showed that using an appropriate tool cathode can create ideal flow and electric fields, resulting in better processing. After optimizing, the machining plane arithmetic mean deviation decreased by 43% (from 14.050 μm to 6.045 μm), and the region elevation difference decreased by 52% (from 105.93 μm to 55.17 μm).

## 1. Introduction

With the development of machining technology and improvements at industrial level, the application scenarios for national defense military equipment, civil aviation aircraft, and reciprocating space aircraft are becoming extremely complicated [1]. The rapidly developing aerospace technology demands suitable materials for weight reduction and structural optimization of airspace engines and fuselage parts [2]. Aluminum alloys have the advantages of high strength, low density, and good corrosion resistance [3]. Adding auxiliary elements such as copper, magnesium, and zinc, can produce two-series, six-series, and seven-series aluminum alloys with high toughness, heat resistance, and damage resistance [4].

Generally, the 2219 aluminum alloy has a good cutting performance. However, for some hard-to-machine parts such as fairing, grid fins, and fuel storage tanks [5] that have a high integration degree, light weight, small wall thickness, and a large margin removal of blanks [6], the process of mechanical cutting can cause chatter, residual stress [7], deformation, and other behaviors [8]. This can lead to processing defects, reduced production efficiency, and increased manufacturing costs [9]. Electrochemical machining technology is not limited by the physical and mechanical properties of materials and tool electrodes [10] such as the cutting force and cutting heat, and can effectively control the deformation of parts [11]. It is widely used in the aerospace field and is suitable for processing large-size aluminum alloy parts [12]. To meet the complex machining requirements of such macro-parts and to improve the surface quality, the electrochemical milling of AA 2219 is worth studying.

To accurately predict the ECM results and improve the machining quality, ECM processing simulations have been the focus of many researchers. Yu simulated and analyzed gas–liquid two-phase flow and temperature fields in a counter-rotating ECM, predicted the results of local electrochemical machining, and machined a convex casing to meet requirements [13]. Skoczypiec performed a electrochemical–electro discharge combination machining computer simulation, oversaw a process of mathematical modeling, and developed a software of combined sequence for ECM operations [14].

He studied the high-flow-zone distribution in a flow-field simulation of the TiB2/7050 Aluminum Matrix Composite ECM machining process, and demonstrated that a tool with a concave arc surface at the end can improve the bottom-surface flatness of the machined groove. However, it reduced the material’s removal rate [15]. Liu proposed a method for controlling the flow field, using the tool-cathode design, during machining to reduce stray corrosion on the workpiece surface outside the processing area. Brilliant silvery grooves were obtained using a tool with an appropriate downward slope angle and an ideal two-phase flow field [16].

Flying electrochemical milling (FECM) is a method that uses an electrode with an opening at the bottom and is installed on a liquid supply electric spindle. The electrolyte is sprayed into the machining gap, and the electrode is placed above the surface to be machined without contact [17]. This method is beneficial for the removal of electrolytic by-products using the three-dimensional motion and rotation of the cathode, and a complex three-dimensional profile can be easily machined [18]. However, there is a lack of relevant theoretical and experimental research on flying electrochemical machining of aviation aluminum alloys.

In this study, we propose two optimization criteria for the tool-cathode design of an internal electrolyte spray tool using flow and electric-field-simulation models. The correlation between the plane arithmetic mean deviation of the machined surface and the regional elevation difference from the simulation results was verified experimentally. By optimizing the tool cathode, we reduced the 20 mm × 20 mm plane arithmetic mean deviation by 43% and the region elevation difference by 52%, and proposed further research plans in the future, which lays the foundation for further research on ECM experiments for AA 2219.

## 2. Flow and Electric Simulation, Analysis, and Optimization

**Figure 1:**
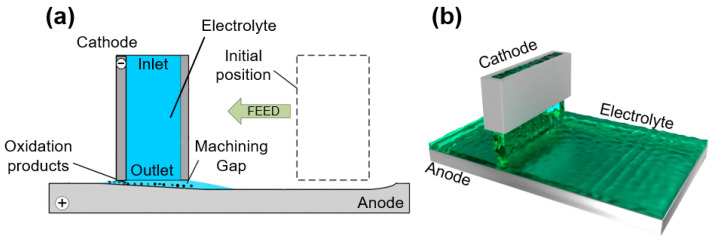
A schematic of flying electrochemical milling with a rectangular tool cathode. (**a**) Two-dimension; (**b**) three-dimension.

Figure 1 shows the formation process of flying electrolytic milling using a rectangular tool cathode with a through hole. The electrolyte is sprayed into the machining gap between the tool and workpiece from top to bottom. During processing, hydrogen bubbles are generated on the cathode surface of the tool, the anode material of the workpiece is removed in the form of ions, and insoluble oxidation by-products are formed. These by-products and Joule heat are discharged from the machining gap using high-speed electrolyte flow to ensure the stability of the flying electrolytic milling process.

### 2.1. Flow-Field-Simulation Model

**Figure 2:**
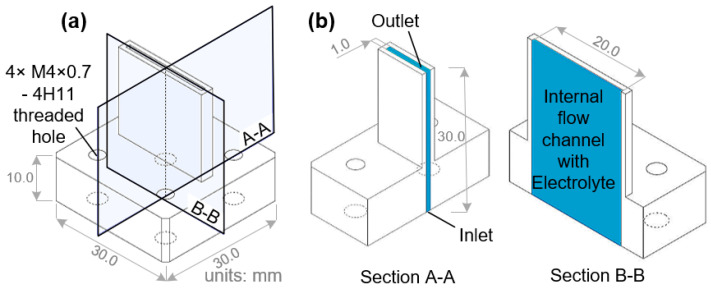
Diagram of the initial rectangular tool cathode A with orthogonal sections. (**a**) Three-dimensional profile of tool A. (**b**) Profiles of orthogonal cross sections: A-A and B-B.

The uniformity and controllability of the flow and electric-field distributions of the machining gap have a significant impact on the machining accuracy and surface quality of the workpiece, which are determined by the inner structure and outlet of the tool cathode. Therefore, in the flying electrolytic milling process, the structure of the tool cathode can be designed to improve the stability and processing quality. Figure 2 shows the criteria dimensions, structural and cross-sectional diagrams of the original undesigned tool cathode A. All tool cathodes used next in this study were made using 304 stainless steel.

**Figure 3:**
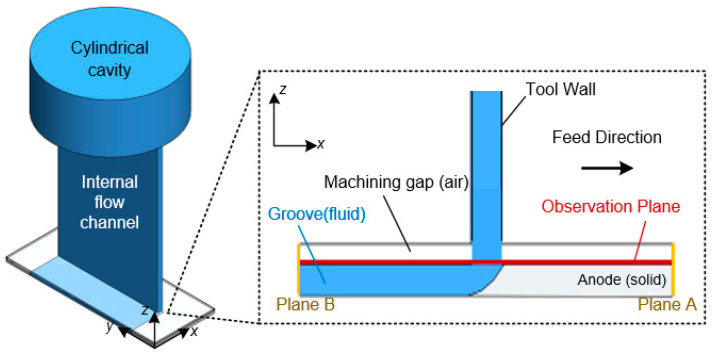
Geometric model of the flow-field simulation. Plane A/B represent the two planes for monitoring liquid mass flow rate.

During processing, the electrolyte flows into the internal flow channel, through the machining gap, and out from the groove surface. A 3D model of the electrolyte flow-field simulation in the machining process, using the original tool cathode, is shown in Figure 3. The electrolyte flows into the cylindrical cavity from the pipe through pumping further into the internal flow channel of the tool cathode, and it is ejected from the bottom of the tool cathode to the workpiece surface. A thin layer of air exists between the bottom of the tool cathode and the surface of the workpiece, which is the machining gap in the actual machining process. The left and right yellow lines represent the two planes used for monitoring the liquid flow. In the simulation, the gray area is the solid phase, the blue and processing gap areas are the liquid phase. Table 1 lists the boundary condition settings for the flow-field simulation. Because the tool cathode was not rotated along the *Z*-axis in this study, the calculation results had little correlation with time; therefore, a steady-state simulation was chosen for simulating the flow field [19].

#### 2.1.1. Optimizing the Internal Flow Channel in the Tool Cathode

**Figure 4:**
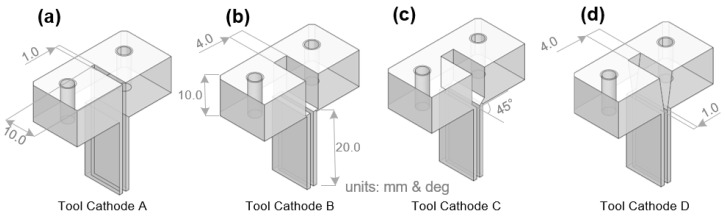
Sectional view along the centerline for four tool cathodes with different internal flow channel designs. (**a**) Tool A. (**b**) Tool B. (**c**) Tool C. (**d**) Tool D.

**Figure 5:**
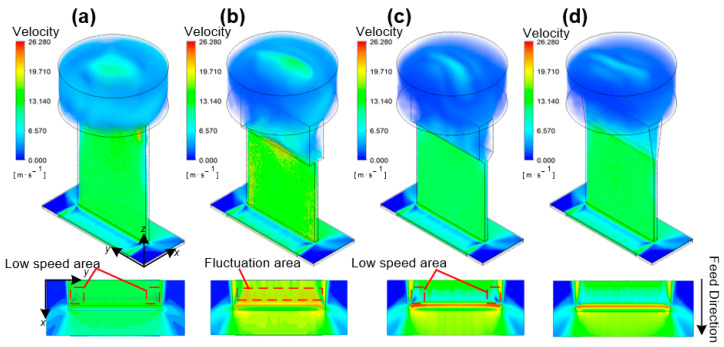
Electrolyte velocity distribution in the machining gap using tools with different internal flow channels. The velocities are all characterized using a unified scale. (**a**) Tool A. (**b**) Tool B. (**c**) Tool C. (**d**) Tool D.

To explore the influence of the internal flow channel shape on the flow-field distribution in the machining gap, a geometric model was established according to the parameters in Section 2.1; boundary conditions were set, and simulation calculations and post-processing were performed. Four types of rectangular tool cathodes with different internal flow channel structures were designed, as shown in Figure 4. Tool cathode A is undesigned without an internal channel structure; tool cathode B has a two-layer rectangular inner channel with a mutated shrunken cross-sectional area. Tool cathode C is updated with a 45° oblique transition section from the upper to the lower region based on tool B; Tool cathode D transforms the rectangular flow channel into a trapezoidal flow channel. Figure 5 shows the electrolyte flow rate distribution in the machining gap when different tools were used.

The ANSYS Fluent module was used to simulate four tool cathodes with different inner channel structures, under the same initial simulation parameters and boundary conditions. To feel the changes in the fluid velocity caused by the internal flow channel structure more intuitively, the velocity in the figures is characterized using a unified scale, and the unit is m·s^−1^.

For tool cathode A without an internal channel structure, the velocity mutation disorder region can be clearly observed when the electrolyte enters the rectangular internal channel from the cavity. During the simulation, although the results converged, the flow velocity distribution inside the rectangular channel was still uneven and a high center and low end trend was observed. On the observation plane of the machining gap, the area marked with red dashed lines in Figure 5a is the low-flow-rate area of the electrolyte, which makes it difficult to discharge the electrolytic by-products. The Material Removal Rate (MRR) in the low-flow-rate area was lower than that in the high-flow-rate area at the same time. Therefore, the internal flow channel of tool cathode A must be further optimized.

Tool cathode B had a two-layer rectangular inner channel with a mutated shrunken cross-sectional area. The electrolyte first entered the upper rectangular channel from the cylindrical cavity and then flowed further into the lower rectangular channel. At the upper level, the average flow velocity of the electrolyte was low, whereas at the lower level, it significantly increased. However, because of the mutation in the cross-sectional area of the flow channel, the average flow velocity at the bottom outlet could not reach a uniform and stable state at the processing gap. As shown in Figure 5b, on the observation plane (Z = 2.2 mm), the flow field at the machined groove exhibited multiple velocity fluctuations, which are marked with dashed red lines. Such irregular disturbances also affect the other end of the outlet, causing untimely discharge of the electrolytic by-products generated by the ionization channel, which leads to poor stability and reduced surface consistency of the workpiece.

Cathode C was updated with a 45° oblique transition section from the upper to the lower region based on tool B. The flow velocity in the lower region exhibited good uniformity without obvious fluctuations. On the observation plane, the high-flow-velocity zone was distributed continuously, with a highest velocity of 21.28 m/s, and the flow-field distribution was stable in the feed direction of the outlet. The simulation results showed that adding an oblique transition section to the inner channel can significantly improve the uniformity of the overall Z-velocity distribution and prevent disturbances caused by sudden velocity changes. However, low-velocity-flow areas can be observed at the locations marked with the dashed red line in Figure 5c. The turbulence in the lower channel region is affected by the sidewall of the tool cathode, resulting in a decrease in the fluid velocity near the walls on both sides.

Based on cathode C, the tool cathode D transforms the rectangular flow channel into a trapezoidal flow channel, which realizes a step-by-step transition for the electrolyte entering the upper flow channel from the cavity. In the upper channel, the electrolyte flow velocity gradually increases over the entire Z range. In the lower channel, the flow velocity of the electrolyte exhibited good uniformity without evident high- or low-flow-velocity areas. In the observation plane, the high-flow-velocity area was continuous. The minimum electrolyte flow velocity was at the rear end of the outlet. The flow-field distribution in the front section of the outlet was stable with an average flow velocity of 16.96 m/s, which was the maximum value among the four tool structures, meeting the requirements for high-speed fluid replenishment and scouring in the processing area. In summary, from the perspective of the average flow-velocity distribution of the electrolyte and flow-field uniformity in the front section of the outlet, optimizing the internal flow channel can significantly improve the flow-field performance compared to the original cathode, and plays a positive role in actual processing.

#### 2.1.2. Optimization of the Bottom Outlet Structure of the Tool Cathode

In the electrolytic milling process of a workpiece with a tool cathode, most research has been focused on the outlet layout influence of the cylindrical tool cathode on the flow-velocity distribution in the machining gap. Researchers have improved the uniformity of the flow-field distribution in the machining gap by optimizing the layout of the outlet hole at the bottom. Hansong Li from the Nanjing University of Aeronautics and Astronautics performed a series of studies on the electrochemical machining of difficult-to-machine metal materials through optimizing the following: the design of a cylindrical tool cathode with rounded corners at the bottom, the layout of the outlet at the bottom of the cylindrical tool cathode, and the arc shape at the bottom of the tool cathode. In this section, based on the FLUENT flow-field simulation, the tool structure for optimizing the electrolyte flow-field distribution in the machining gap is further explored. The bottom-outlet shape of the rectangular tool cathode is considered as the independent variable.

**Figure 6:**
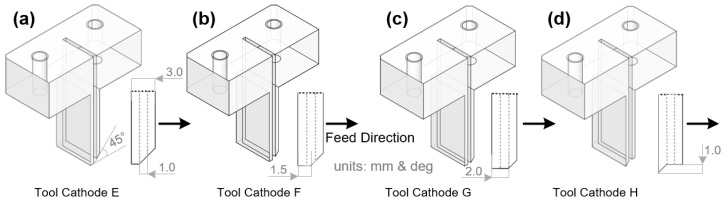
Sectional view along the centerline and enlarged outlet diagram for four tool cathodes with different outlet designs. (**a**) Tool E. (**b**) Tool F. (**c**) Tool G. (**d**) Tool H.

After the exploration in Section 2.1.1, four types of rectangular tool cathodes with different bottom-outlet structures were designed, as shown in Figure 6. Cathode E cuts the entire rectangular outlet hole and a rake face at a 45° angle on the feed direction side, whereas cathode F cuts 1/2 of the rectangular outlet hole and a rake face at a 45° angle on the feed direction side. Cathode G excised the entire rake face at a 45° angle on the feed direction side without changing the shape of the rectangular outlet hole. Cathode H formed a 45° sharp-angle bump on the major back face without changing the rectangular-outlet hole or rake face. The purpose of optimizing the bottom-outlet shape is to obtain a better distribution of the bottom electric and flow fields so that more electrolytes flow to the surface to be machined in the feed direction and to reduce the stray corrosion of the machined surface caused by a splashing electrolyte and the electric field.

**Figure 7:**
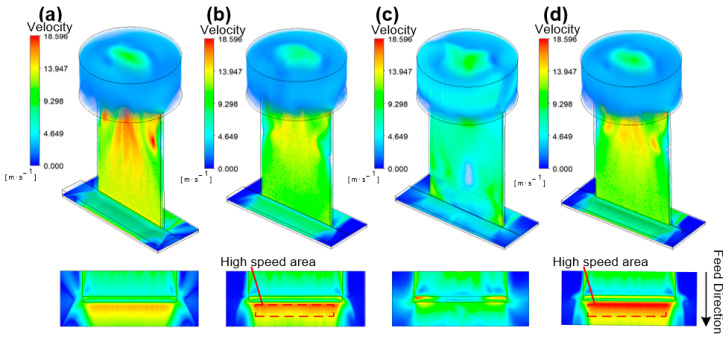
Electrolyte velocity distribution in the machining gap using tools with different outlet designs. (**a**) Tool E. (**b**) Tool F. (**c**) Tool G. (**d**) Tool H.

The ANSYS Fluent module was used to simulate four different outlet-structure tool cathodes under the same initial liquid inlet and boundary conditions, and the simulation results are shown in Figure 7. Simultaneously, the liquid mass flow through planes A and B under a steady state was also monitored in different simulation results to characterize the liquid distribution on the feed side and machined processing side, as shown in Table 2.

To feel the changes in fluid velocity caused by the internal-flow-channel structure more intuitively, the velocity in the figures is characterized using a unified scale, and the unit is m·s^−1^. The flow-field distribution shows that after the electrolyte is flushed down the inner flow channel, it collides with the surface to be machined and scatters in two directions. As shown in Table 2, the mass flow distributions before and after the tool-cathode feeding in the control group were more uniform. 

For the tool cathode E, the mass flow rate of the electrolyte on the machining side accounted for 55.9% of the total liquid output. The mass flow rates of the electrolyte and on the front side of the feed increased by 30% and 44%, respectively, compared to the control group of the tool cathode without the outlet-structure design. 

The mass flow rate of the electrolyte on the side of the tool cathode F accounted for 58.0% of the total liquid output, and it was reduced by 10% compared to that of the tool cathode in the comparison group without the structural design of the liquid outlet; however, the mass flow rate ratio on the front side of the feed was increased. 

The mass flow rate in front of tool cathode G was significantly different from the mass flow rate in the back of the tool cathode G. The mass flow rate of the electrolyte on the machining side accounts for only 33% of the total liquid output. Compared to the tool cathode in the control group, the total mass flow rate of the electrolyte was reduced by 19%, and the mass flow rate in front of the feed was decreased by 17%. 

The total liquid output of tool cathode H increased by 31.6% and the mass flow rate of the electrolyte on the machining side accounted for 52.7% of the total liquid output, which was the best performance among the four groups. It can also be seen from Figure 7d that, affected by the structure of the bottom end of tool cathode H, more electrolyte flows to the surface at a higher flow rate to be machined in the feed direction, and the bottom structure of tool cathode H can optimize the distribution of the electrolyte flow field in the machining gap.

### 2.2. Electric-Field-Simulation Model

In flying electrolytic milling, the tool cathode not only constrains the flow field and provides the electrolyte channel, but also forms a current path with the surface of the workpiece to be machined. The structure of the tool-cathode outlet and current-density distribution on the machining surface significantly influences the localization of machining, surface roughness, and other important indicators.

The relationship between the current density i in the machining gap and electric-field intensity E is derived from Ohm’s law [20]:

(1) i=κE

where, *κ* is the conductivity of the electrolyte.

The relationship between the anode electrochemical-dissolution rate $v_{a}$ on the workpiece surface and the current density *i* is described using Faraday’s law [21]:

(2) $$v_{a}=\eta (i)\omega i$$

where, *η* is the current efficiency and *ω* is the volumetric electrochemical equivalent of the anode material of the workpiece.

From these two equations, it can be inferred that, when the electrolyte conductivity does not change, the current density of the workpiece anode surface is proportional to the electric-field strength and material-dissolution rate.

Therefore, the electric-field simulation model in this section was established to explore the influence of different tool-cathode bottom structures on the surface current-density distribution between the machining gaps of the workpiece. The geometric structure of the electric–field model is consistent with the flow-field model described in Section 2.1. The electrode gap between the bottom of the tool cathode and surface of the workpiece was 0.3 mm. The outlet at the tool-cathode bottom measured 20 mm × 1 mm, and the overall dimensions of the tool-cathode bottom was 22 mm × 3 mm. The dimensions of the enveloping air domain at the tool-cathode bottom were set as 32 mm × 5 mm × 5 mm. The initial and other boundary conditions of the electric-field simulation are listed in Table 3.

To simplify the calculation process, the following electric-field simulation model was assumed [22]:

(1) The electric field in the processing area was regarded as a constant electric field.
(2) The cathode surface of the tool and anode surface of the workpiece to be machined were regarded as having different equal-potential surfaces.
(3) The conductivity of the electrolyte does not change with the processing process, which follows isotropy, and is always equivalent to the conductivity of a clear electrolyte in the nonprocessing state.

**Figure 8:**
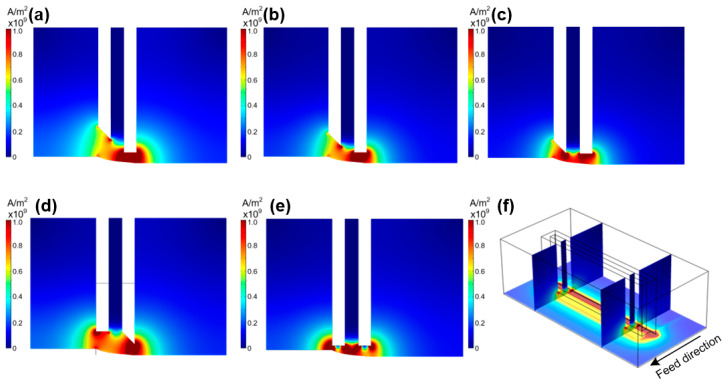
Electric-field-density distribution on the YZ plane using tools with different outlet design. (**a**) Tool E. (**b**) Tool F. (**c**) Tool G. (**d**) Tool H. (**e**) Original undesigned Tool A. (**f**) Three-dimensional profile of the Tool H electric field.

Because the electric-field distribution is only affected by the structure of the outlet at the bottom of the tool cathode, only the four tool cathodes E/F/G/H in Section 2.1.2, compared to the undesigned control group tool cathode A, were selected for electric-field simulation. The simulation results are shown in Figure 8, where (a–d) correspond to the tool E/F/G/H, (e) corresponds to the initial tool cathode A at the bottom without the design, and (f) is the 3D schematic of the final selected electric-field distribution.

For the control group and the initial tool cathode A in Figure 8e, two obvious high-potential points can be observed on the rake face at the bottom of the tool cathode. There are two other points of high current density on the flank side. The high-current-density points were distributed on the sharp corners of the tool cathode; however, they were not uniformly distributed on the bottom surface of the entire tool-cathode outlet. This uneven potential distribution indicates that continuous local large-current channels are generated during processing, which can easily cause local short circuits of current and damage the tool electrodes and machined surfaces.

In Figure 8a, tool cathode E excised the entire rectangular outlet and front cutter face at a 45° angle. Because the distance from the tool cathode to the machining surface increased on the rake face, it is evident that the current is concentrated in the sharp corner structure on the feed side. There is a clear high-current-density region on the bottom surface of the tool cathode and a single high-current-density region is conducive to the formation of a stable electronic channel to ensure the stability of the machining process.

Tool cathode F excised 1/2 of the rectangular outlet hole at a 45° angle from the front cutter face on the feed direction side. This design reduced the distance from the front cutter face to the machining surface. Therefore, compared to Figure 8a, there are more obvious points of high current density at the sharp corners of the front tool cathode B owing to the electron tip-aggregation effect. This leads to the formation of unstable electronic channels between the sharp corners and metal surface during the machining process, which interferes with the stable machining of the groove in the rear-tool surface area.

Figure 8c shows a tool cathode with the entire rake face cut at a 45° angle on the feed direction side and no other design for the outlet hole. As can be seen from the figure, owing to the tip-aggregation effect of electrons, there are two obvious points of high current density on the tool-cathode bottom surface, which indicates that there are still two high-current-density distribution areas on the machined surface that can easily cause stray corrosion on the machined surface.

Tool cathode H formed a sharp-angle bump at an angle of 45° on the back tool face without changing the rectangular outlet hole and rake face. As shown in Figure 8d, there is an obvious point of high current density at the sharp corner of the back tool face. Although, there is also a high-current-density area, and because of the distance between the front tool face and the machined surface, the influence on the machined surface is far less than that of the back tool face, as shown in Figure 8f.

### 2.3. Analysis for Simulation

Based on the above simulation of the electric- and flow-field distributions of the tool cathodes, this section concludes that by optimizing the internal flow-channel structure of the tool cathode the electrolyte flow can be prioritized in the feed direction by the internal flow-channel structure with a trapezoidal flow channel and step-by-step transition compared to the initial tool cathode. The mass flow rate of the electrolyte increased simultaneously. By optimizing the bottom outlet of the tool cathode, tool cathodes E/H can form a stable single high-current-density region on the machined surface in terms of the electric-field distribution. Compared to the original tool cathodes A and E, the flow field formed by the bottom structure of tool cathode H was more high speed, uniform, and stable. The simulation results in this section can be used to guide further experiments.

## 3. Experimental Verification 

### 3.1. Experimental Set up

**Figure 9:**
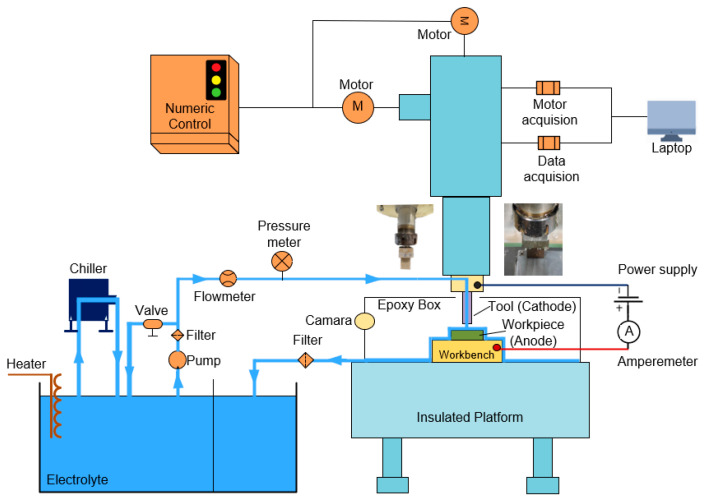
Schematic of the ECM System.

The ECM system used in the electrolytic milling experiment is shown in Figure 9, including the computer program control, data acquisition, motion control, electrolyte circulation, and power supply. The tool material was 304 stainless steel (manufactured based on Chinese National Standard GB/T 20878-2015), and the anode material was AA 2219 (manufactured based on Chinese National Military Standard GJB 2622A-2008) [23,24]. A DC power supply was used as the input (ITECH IT-M100, Nanjing, China), and the specification parameters of the power supply are shown in the Table 4. The tool cathode and each piece of the anode material were cleaned and dried using an ultrasonic cleaner before machining to ensure that every surface was free of impurities that would interfere with ECM. After each experiment, the machined surface was ultrasonically cleaned and blow-dried to remove the electrolytic by-products and electrolytes attached to the surface.

**Figure 10:**
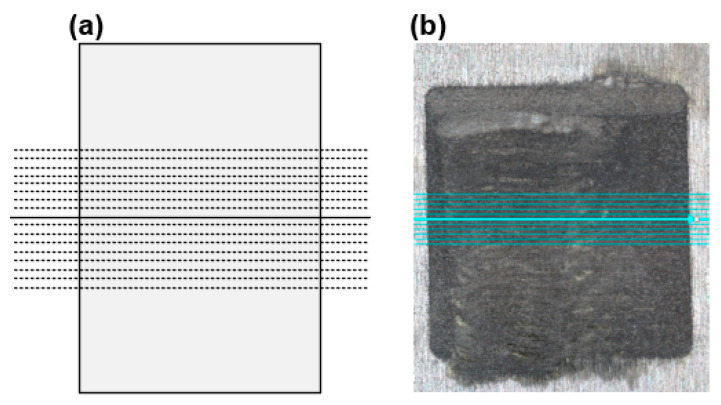
(**a**) Contour measuring method. The solid line at the center represents the vertical direction center, and the dashed lines on the upper and lower sides represent other measurements taken with averaging. (**b**) Physical image of measuring.

To explore the effect of the ECM process using the two different types of designs, experiments were performed using the same machining parameters with different cathodes. The machining parameters are listed in Table 5. The 3D contour shape of the machined workpiece was measured using a wide-area three-dimensional measurement system (KEYENCE VR-5000, Osaka, Japan). The horizontal contour lines were measured using the method shown in Figure 10. In the cross-section of the vertical center, eight horizontal lines at a distance of 200 µm from both sides were recorded and the horizontal contour measurement results of all the lines were averaged. 

The quality of the machined surface is defined using its plane arithmetic mean deviation (*S_a_*) and region elevation difference (*S_z_*), where *S_a_* represents the arithmetic mean of the absolute of the ordinate values within a definition area, and *S_z_* represents the sum of the maximum-peak-height value and the maximum-pit-height value within a definition area. *S_a_* and *S_z_* are defined in ISO-25178-2:2012—Section 4: field parameter definitions, both directly measured using KEYENCE Analyzer (Version VR-5000 Software) [25]. The analysis and measurement method for *S_a_* and *S_z_* is as follows: all points in the 3D profile of the groove with a height difference of more than 20 μm from the datum plane are selected as the definition area, and *S_a_* and *S_z_* are obtained through calculation using the following equations [26]:

(3) $$S_{a}=\frac{1}{A}\iint_{A}\left|z\left(x,y\right)\right|\;dxdy$$

(4) $$S_{z}=\left|min\left(z(x,y)\right)\right|+\left|max\left(z(x,y)\right)\right|$$

### 3.2. Effect of the Internal Flow Channel on the Experimental Results

**Figure 11:**
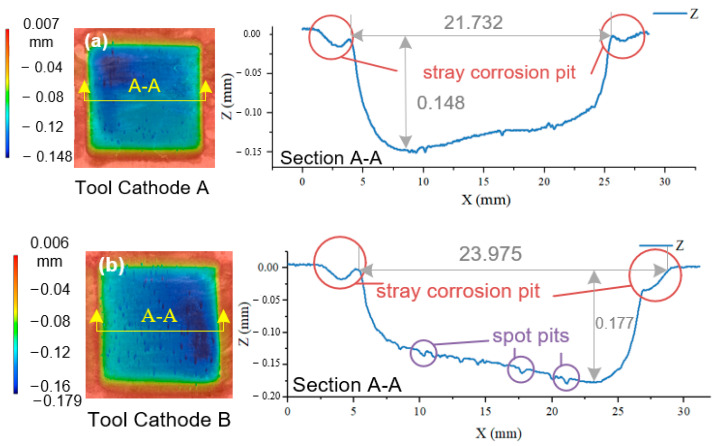

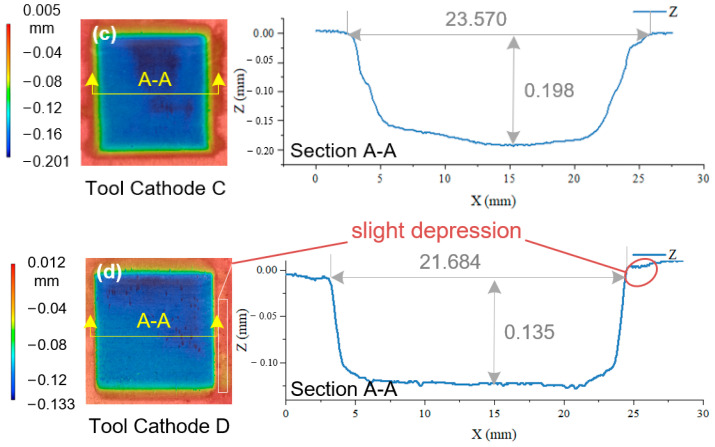
AA 2219 single-groove 3D contour shape and horizontal contour lines obtained using ECM with different internal-flow-channel tools under the same parameters. (**a**) Tool A. (**b**) Tool B. (**c**) Tool C. (**d**) Tool D.

The internal-flow-channel design is an important factor that affects the ECM process. Figure 11 shows the change in the workpiece surface topography and profile curve with changes in the internal-flow-channel design.

As shown in Figure 11, the design of the internal flow channel of the tool cathode has a significant influence on the uniformity of the machined surface and the degree of stray corrosion. Figure 11a shows the surface obtained using tool cathode A. The cross sectional shape of the bottom surface has a large deviation, and is close to an uneven slope, and the difference in the machining depth is approximately 0.05 mm. Meanwhile, there was severe stray corrosion on both sides of the groove, as indicated by the red circles. This side corrosion causes serious cutter marks while destroying the finished surface.

Figure 11b shows the surface machined using tool cathode B. In addition to the uneven shape of the bottom surface and the stray corrosion, there are many spot pits (purple circles) on the machining surface, which correspond to the flow-field-analysis results in Section 2.1.1, indicating that the machining surface of tool cathode B has areas with serious flow-rate fluctuations.

**Figure 12:**
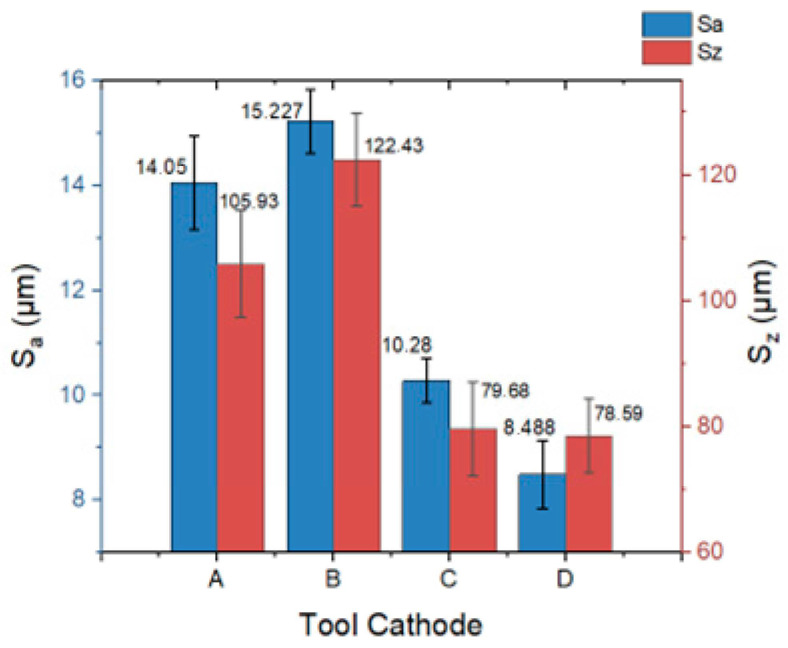
Plane arithmetic mean deviation (*S_a_*) and region elevation difference (*S_z_*) with different tools under the same parameters. Error bars represent the standard deviation of the five individually measured results.

In contrast, the machining results in Figure 11c,d show the positive effects of a uniformly distributed flow field on the processing quality of the machined surface and the inhibition of stray corrosion. Compared to (a) and (b), the flatness of the bottom surface in Figure 11c is significantly improved, and the bottom outline no longer has an obvious difference in machining depth. As shown in Figure 11d, there was only a slight depression caused by stray corrosion on the right side of the groove, and the flatness of the bottom plane was further improved. The *S_a_* and *S_z_* distributions for the four results are shown in Figure 12.

As shown in Figure 12, the flow-field distribution on the machined surface gradually improved with a change in the cathode. When tool cathode B was used, both *S_a_* and *S_z_* reached their maximum values (15.227 ± 0.610 μm, 122.43 ± 37.35 μm). The homogenization of the flow-field distribution makes the flow rate per unit time constant, and the products in the processing gap can be stably discharged, which is conducive to improving the surface quality. When tool cathode D was used, both *S_a_* and *S_z_* reached their minimum values (8.488 ± 0.647 μm, 78.59 ± 5.96 μm).

### 3.3. Effect of Bottom-Outlet Structure on the Experimental Results

**Figure 13:**
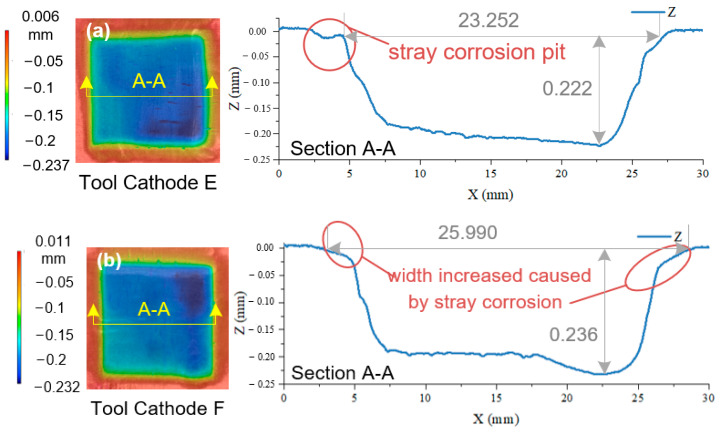

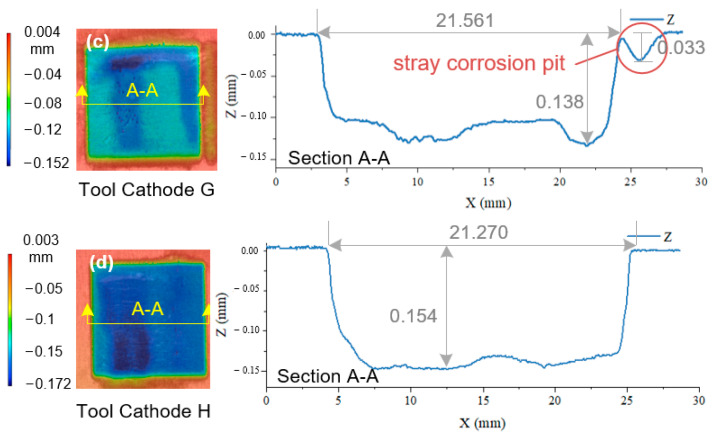
AA2219 single-groove 3D contour shape and horizontal contour lines obtained using ECM with different bottom-outlet-structure tools under the same parameters. (**a**) Tool E. (**b**) Tool F. (**c**) Tool G. (**d**) Tool H.

Figure 13 shows the change in the workpiece surface topography and profile curve with a change in the bottom-outlet-structure design. It should be noted that because only the outlet structure was designed in this section, the flow field in the actual experiment was occasionally in an unstable state owing to the influence of the internal flow channel. Therefore, the experimental results obtained in this section are consistent with those in the previous section and do not constitute a progressive relationship.

The influence of the bottom-outlet shape on the machining quality was analyzed according to the results shown in Figure 13a. The surface was obtained using tool cathode E. There is a shallow pit caused by the edge effect on the left side of the groove, and the bottom plane is slightly inclined with a height difference of 0.05 mm. Figure 13b shows the surface processed using tool cathode F. Both sides of the groove were significantly affected by the edge effects, and the pits were integrated with the groove. Such machining results make it difficult to accurately control the size of the machining area. The bottom surface in Figure 13c has two significantly deeper concaves caused by an uneven flow field. When the local electrolyte flow speed is too high, more metal atoms complete the electron exchange per unit time, resulting in deeper removal depths.

**Figure 14:**
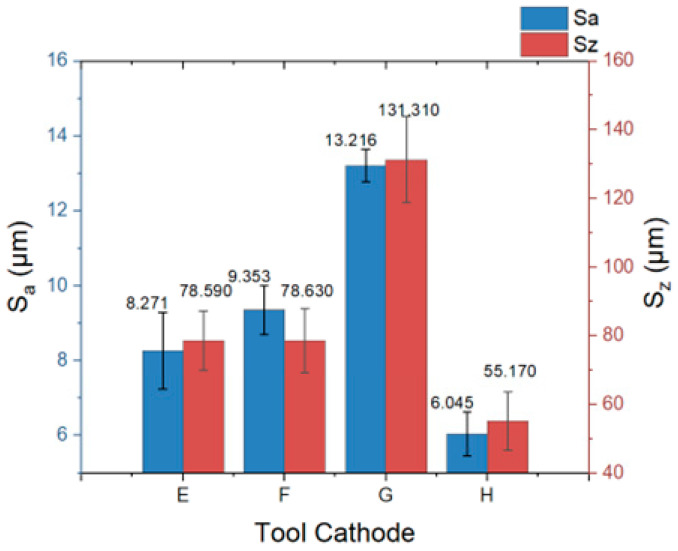
Plane arithmetic mean deviation (*S_a_*) and region elevation difference (*S_z_*) with different tools under the same parameters. Error bars represent the standard deviation of five individually measure results.

The flow-field-simulation results for tool cathode H were the best among the four designs. As can be seen from the processing results in Figure 13d, both sides of the groove are not subjected to stray corrosion, the localization of the processing is very good, and the angle between the wall surfaces on both sides and the reference plane is close to 90°, which verifies the conclusions in the previous sections. Forming a uniform flow field and stable single region of high current density on the machined surface is vital to obtaining better quality. From the perspective of the bottom-surface topography, the processing did not enter a stable state at the beginning of the machining stage; however, with the stability of the tool feed state, the flatness of the bottom surface was improved and maintained, indicating that the flow field in the machining area reached a stable equilibrium state. The *S_a_* and *S_z_* distributions for the four results are shown in Figure 14.

As shown in Figure 13, when the tool cathode G was used, both *S_a_* and *S_z_* reached their maximum values (13.216 ± 0.430 μm, 131.31 ± 12.60 μm). While with the tool cathode H, both *S_a_* and *S_z_* reached the minimum values (6.045 ± 0.582 μm, 55.17 ± 8.56 μm). Compared to the original plane processed using tool cathode A, *S_a_* and *S_z_* decreased by 43% and 52%, respectively. 

## 4. Conclusions

To study the inner-jet electrochemical milling of AA 2219, two types of tool-cathode-design schemes were proposed to optimize the internal flow channel and change the structure of the bottom outlet. A simulation analysis of the flow and electric fields was used in the experiment, and an ECM experiment was conducted to verify the results. Several 20 mm × 20 mm surfaces were machined. The main conclusions of this study are as follows.

(1) During the ECM process, unstable flow-field disturbances or easily changed high-current-density regions cause local short circuits or poor uniformity of the machining surface. Therefore, optimizing the outlet structure should be aimed at “forming a stable uniform flow field with priority feed direction under the outlet of the tool cathode” and “forming a stable single region of high current density on the machined surface”.
(2) Owing to the electron tip-aggregation effect, the current density at the two sides of the rectangular tool is higher than that in the middle, resulting in stray corrosion and side effects on both sides of the groove, resulting in a shallow pit that affects the width of the machining plane, such as the result of tool cathode F in Figure 12 By confining the electric-field distribution of the rectangular cathode, stray corrosion can be effectively reduced and a good localized machining surface can be obtained.
(3) The two methods for optimizing the tool cathode proposed in this study produced positive effects to varying degrees. Using the optimized cathode H machining, compared to the original tool A, the plane arithmetic mean deviation decreased by 43% (from 14.050 μm to 6.045 μm) and the region elevation difference decreased by 52% (from 105.93 μm to 55.17 μm). The experimental results match the simulation results, indicating that the two design methods can be combined to further improve surface quality.

## Figures and Tables

**Table 1:** Flow-field-simulation parameters and boundary conditions.

Parameter	Value
Inlet pressure (MPa)	0.2
Outlet pressure (MPa)	0
Side and bottom gap (mm)	0.3
Machining groove depth (mm)	0.5
Detailed dimensions of the outlet (mm)	20 × 1

**Table 2:** Feed direction and reverse mass flow rate meter for tools A/E/F/G/H.

Tool Cathode	Mass Flow Rate on Plane A (Feed Direction) ( $kg\cdot s^{-1}$)	Mass Flow Rate on Plane B (Reverse Direction) ( $kg\cdot s^{-1}$)
A (undesigned)	0.091	0.089
E	0.131	0.103
F	0.094	0.068
G	0.049	0.098
H	0.125	0.112

**Table 3:** Electric-field-simulation parameters and boundary conditions.

Parameter	Value
Current-simulation model	Primary current-distribution model
Tool-cathode potential (V)	0
Metal anode potential (V)	50
Machining groove depth (mm)	0.5
Machining gap (mm)	0.3
Detailed dimensions of the outlet (mm)	20 × 1
Cathode Type	E/F/G/H/A

**Table 4:** Specification parameters of the programmable DC power supply.

Parameter	Value
Output Voltage (V)	0~80
Output Current (A)	0~450
Output Power (W)	0~15,000
Voltage Line Regulation (±% of Offset)	≤0.01% FS
Voltage Load Regulation (±% of Offset)	≤0.02% FS
Voltage Programming Resolution (V)	0.001

**Table 5:** Parameters of the single-groove milling experiment.

Parameter	Value
Applied voltage (V)	40
Feed rate (mm·min^−1^)	40
Electrolyte (wt.%)	20% NaCl
Electrolyte temperature (°C)	20
Electrolyte pressure (Mpa)	0.2
Machining gap (mm)	0.3
Tool cathode	A/B/C/D/E/F/G/H

## Data Availability

The data presented in this study are available on request from the corresponding author.
